# Food and Nutrition Related Concerns Post Lockdown during COVID-19 Pandemic and Their Association with Dietary Behaviors

**DOI:** 10.3390/foods10112858

**Published:** 2021-11-18

**Authors:** Zhongyu Li, Ai Zhao, Jufang Li, Yalei Ke, Shanshan Huo, Yidi Ma

**Affiliations:** 1 Vanke School of Public Health, Tsinghua University, Beijing 100091, China; zli132@jhmi.edu (Z.L.); nmgljf109@163.com (J.L.); 2 Johns Hopkins Bloomberg School of Public Health, 615 N Wolfe St., Baltimore, MD 21205, USA; 3 School of Public Health, Peking University, Beijing 100191, China; keyalei@pku.edu.cn (Y.K.); tuanzishan@pku.edu.cn (S.H.); 1810306135@pku.edu.cn (Y.M.)

**Keywords:** dietary behaviors, food purchases, COVID-19, post COVID-19 lockdown, nutrition, stress, socio-demographics

## Abstract

This study aimed to explore food and nutrition related concerns during the post-lockdown period and their impacts on food shopping, preparation, and eating behaviors. Design: A cross-sectional study was conducted through online questionnaire. We collected data on food and nutrition related concerns, socio-demographic characteristics, and changes in dietary behaviors. Participants: A total of 2267 responses were received from people living in 31 provinces across mainland China and 1994 participants were included in the final analysis. Analysis: Linear and multinomial logistic regression analyses were performed to evaluate the association among food and nutrition related concerns, socio-demographic factors, and dietary behaviors Results: About 56% of participants reported moderate to high levels of concerns related to food and nutrition. Contracting the virus when dining out or through contaminated foods were the top two concerns, followed by overnutrition. Higher levels of concerns were found among people who were older, overweight, or obese, having lower income and education, and living in urban areas, or whose family contained vulnerable individuals. Compared with the pre-COVID-19 period, people who were more concerned about food and nutrition were more likely to report changes in their food purchases and consumption; they were also more likely to eat from individual plates, using serving chopsticks, and separating plates and utensils for raw and cooked foods during the post COVID-19 lockdown period. Conclusion and Implication: Food and nutrition related concerns during the post-lockdown period were prevalent and associated with changes in dietary behaviors. Preventative policies and nutritional guidance should be developed to address these concerns in order to reduce inappropriate dietary behaviors amid public health crises.

## 1. Introduction

The coronavirus disease 2019 (COVID-19)—caused by severe acute respiratory syndrome coronavirus 2 (SARS-CoV-2) (a novel coronavirus)—is responsible for the current pandemic threatening global public health [1]. As of 11 September 2021, there were over 220 million cases and 4.6 million death reported worldwide [2]. Since the emergence of COVID-19 outbreak in December 2019 and the declaration of pandemic by the World Health Organization (WHO) in March 2020, many countries have mandated self-quarantine and large-scale lockdowns to battle against the rapid spreading of the virus. These COVID-19 related measures and restrictions have negatively impacted the economy and forced people to change their lifestyle, which has often led to exacerbated financial and psychological stresses [3,4,5].

Rising levels of concern, stress, and anxiety have sounded the alarm regarding public mental health during the pandemic [6,7]. A systematic review conducted by Vindegaard and Benros showed decline in mental health wellbeing and increases in stress, depression and anxiety among health care workers, patients with preexisting psychiatric disorders, and the general population [8]. Notably, people experiencing a high level of stress and concern have been shown to exhibit unhealthy eating behaviors such as frequent food purchases and snacking, some of which are associated with greater weight gain and poor nutrition during lockdowns [5,9,10,11]. Indeed, COVID-19 lockdowns brought major behavioral changes in eating, grocery shopping, and physical activities. A Polish study revealed that over 40% of participants ate and snacked more often; there were also rises in alcohol consumption and smoking frequency during nationwide quarantine [12]. Other studies have further indicated more sedentary lifestyle [13], changes in grocery shopping behaviors [14,15], and eating in response to stress [16] during COVID-19 related isolation.

Eating is an integral part of daily life. A balanced diet may enhance immunity, prevent excessive inflammation and weight gain and promote healthy gut microbiocidal composition to battle against infections [17,18,19,20,21,22]. Food shopping, preparation and consumption are important dietary behaviors that play critical roles in the physical and psychological wellbeing of individuals; some of those behaviors may also induce long-term consequences in the food industry. Recent shifts in lifestyle and contraction in economic activities due to COVID-19 restrictive measures as well as suspected COVID-19 cases linked to certain food products and packaging have caused confusion and concerns over food safety, food supply, and nutritional status, which may influence dietary behaviors and cause health problems [23,24,25,26].

Despite a growing number of studies on the mental and behavioral impacts of COVID-19 lockdowns in the literature, research into concerns specific to food and nutrition and how they affect dietary behaviors post-lockdown is sparse. This study aimed to understand how COVID-19 related food and nutrition concerns shape food shopping, preparation, and eating behaviors through surveys conducted post-lockdown in mainland China.

## 2. Materials and Methods

### 2.1. Study Design and Participants

We conducted a cross-sectional survey through the Wenjuanxing e-questionnaire platform (Wenjuan xing Tech Co., Ltd., Chansha, China) in August 2020. This study is the second wave of a longitudinal nutrition study initiated in March 2020 (the first survey) [27]. The current survey aims to understand nutrition and diet related issues during and post-COVID-19 pandemic lockdown in mainland China. We used a multistage sampling method to identify potential study participants and ensured recruitment of people living in South, North and Central China where more participants were then reached by a “snowball sampling” method [27].

A total of 2267 Chinese residents responded to the survey. Our study participants were recruited from 31 provinces, covering most geographic regions in mainland China. We embedded attention checking questions (e.g., “Have you answered the questions seriously?” and questions with instructions to give pre-specified answers) to determine the seriousness of respondents; we subsequently removed nonserious participants to improve data quality (*n* = 264). Furthermore, participants living outside mainland China (*n* = 9) were excluded, resulting in a total of 1994 participants’ responses usable for subsequent data analysis.

### 2.2. Data Collection

The e-questionnaire (Supplementary Materials) consisted of 22 questions grouped into three sections: (1) basic socio-demographic information; (2) dietary diversity and food shopping behaviors; (3) specific dietary behaviors and food and nutrition related concerns. Data containing socio-demographic information, food shopping behaviors, and dietary behaviors and concerns were used in this study. There were no missing data on key questions.

To explore grocery shopping and food storage patterns, we asked participants to identify where and how they have been purchasing/obtaining recently consumed foods including cereals; roots and tubers; vegetables; fruit; meat, poultry and offal; eggs; fish and seafood; pulses, legumes, and nuts; dairy products; oils and fats; sugar and honey; and miscellaneous (such as condiments, snacks and beverages). These 12 food items originated from the Household Dietary Diversity Score (HDDS) developed to measure household access to food [28]. Participants could choose from five options (e.g., home storage, in-person grocery shopping, online food shopping, government or community-based food distribution programs, and no consumption), and multiple selections were allowed. We gave one point per food item per option selected. The possible values for each option ranged from 0 to 12. A higher score indicates a higher tendency of this specific food purchasing and storage pattern.

Five questions pertaining to food and nutrition related concerns were asked to participants to rate their levels of concerns on five relevant issues: (1) contracting the virus through contaminated foods; (2) contracting the virus when dining out; (3) food supply shortage; (4) overnutrition; (5) undernutrition. We used the Likert scale and assigned one point to “not at all concerned”, two points to “slightly concerned”, three points to “moderately concerned”, and four points to “extremely concerned”. Total score ranging from 5 to 20 for each participant was calculated, ranked from lowest to highest, and then grouped into one of the three categories: low level of concern (level 1: 5–8), moderate level of concern (level 2: 9–11), and high level of concern (level 3: 12–20). Besides, we asked the participants to report perceived changes (e.g., increase, decrease, or no change) in food consumptions and dietary behaviors, such as frequency of seafood consumption and eating out, in comparison to the same period last year (2019). Other eating and cooking related behaviors such as using serving chopsticks and cooking food thoroughly were also asked in the questionnaire and examined in this study.

### 2.3. Ethics

The questionnaire was filled in anonymously. Informed consent was required prior to the survey by clicking the “agree” option to confirm willingness to participate voluntarily in the survey. The online survey was conducted in full agreement with the national and international regulations in compliance with the Declaration of Helsinki (2000). This study was approved by Tsinghua University Health Research Center Ethics Committee (NO.THUSM/PHREC/2020400-004).

### 2.4. Statistical Analysis

Demographic characteristics were grouped into categories and presented as percentages by three levels of concerns (created by xtile function in Stata). We assessed associations among categorical variables using Chi-squared test. Simple and multiple linear regression analyses were applied to estimate the effects of food and nutrition related concerns on grocery shopping and food storage patterns, whereas simple and multiple logistic regression were applied to estimate those effects on eating and cooking behaviors. To model the effects of food and nutrition related concern on changes in food consumption and dietary behaviors, we used multivariate multinomial logistic regression in which the dependent variable consisted of three categories (increase, decrease, and no change) and “no change” was selected as the base outcome. We also ran “testparm” and “test” to perform Wald tests on selected coefficients from which *p*-values were obtained. In models adjusting for body mass index (BMI), only 1854 subjects who reported both valid weights and heights were included. We used BMI cutoffs for Asians (underweight < 18.5 kg/m^2^, normal 18.5–22.9 kg/m^2^, overweight 23–26.9 kg/m^2^, and obese ≥ 27 kg/m^2^) to determine participants’ weight categories. All statistical analyses were conducted using Stata version 15.1 (StataCorp. 2017. Stata Statistical Software: Release 15. College Station, TX, USA: StataCorp LLC). We considered a *p*-value less than or equal to 0.05 (≤0.05) statistically significant.

## 3. Results

A total of 1994 participants were included in the final analysis. Most study participants’ ages ranged from 18 to 30 (>60%) with 37% identified as male and 83% living in urban areas (Table 1). There were 1854 participants who self-reported weight and height, among whom 52% had normal weight and more than 30% were overweight or obese. Over half of our study participants (58%) had received bachelors’ degrees and 49% had at least one vulnerable individual (e.g., children under the age of 5, elders above the age of 65, and pregnant women) living in the same household. Most participant reported either low (44%) or moderate (42%) level of food and nutrition related concerns with a mean score at 10.6 (± 3.6). Worries about coronavirus infection while dining out received the highest mean score (2.6 ± 0.92) followed by contracting virus through contaminated foods (2.5 ± 0.99), overnutrition (1.91 ± 0.99), shortage of food supply (1.69 ± 0.93), and undernutrition (1.5 ± 0.82). Several socio-demographic characteristics including age, BMI, income, education, geographic region, and presence of vulnerable individuals significantly associate with different concern levels (Table 1). Elders, who are overweight or obese, less educated, and living with vulnerable individuals in rural areas with lower annual income are more likely to report high level of concerns than younger participants who are well educated and living in urban areas with relatively high annual income and normal BMI (Table 1).

We observed that in-person grocery shopping at local markets was the preferred method of purchasing/obtaining food among our study participants (mean score = 7.9, ±3.4) followed by home storage (4.0, ±3.5) and online grocery shopping (2.1, ±3.2) in the most recent week. Though very few people relied on food distributed through community or government-based programs in the study sample, high level of concerns appeared to associate with an increase in obtaining food from these programs (Table 2). A high level of food and nutrition related concerns has also been found to associate with higher odds of eating from individual plates, using serving chopsticks and separating plates and utensils for raw and cooked foods (Table 3).

Regarding the perceived changes in the frequency of food consumption and dietary behaviors compared with the same period in the previous year (2019), we detected an overall increasing trend for shopping food online and cooking and eating at home. Apart from that, many participants had reduced their consumption of seafood, raw food, and imported fresh produce (Table 4). Study participants who were highly concerned about food and nutrition related issues were more likely to report a change in their food consumptions and dietary behaviors than those who had low level of concern. People who were moderately or highly concerned decreased their consumption of seafood, raw food, and imported fresh produce. In addition, after adjusting for gender, age, education, BMI, annul household income, geographic regions (urban or rural), and meal regularity, a high level of concern was positively associated with either an increase or a decrease in purchasing food online and from farmers’ markets, and cooking and eating at home (Table 4).

## 4. Discussion

In this study—among the first reporting food and health related concerns—we observed that concerns exist in the Chinese population even in the post-lockdown era; we found strong associations between several socio-demographic characteristics and a higher level of concern. Moreover, people who were highly concerned about food, nutrition, and health during the current pandemic were more likely to report changes in their dietary behaviors including how or where foods were purchased and cooked, and the frequency of consumption. Results from our study warrant further investigations into the mental and behavioral impacts of the COVID-19 pandemic that are likely to extend beyond lockdowns for years after the pandemic is contained.

### 4.1. Associations between Sociodemographic Characteristics and Concern Levels

Recent studies have acknowledged the exposure to COVID-19 related stressors and mental health deterioration as well as their impacts on dietary behavioral changes during lockdowns taking place in many countries [4,29,30,31]. Our study added new information to the existing body of literature by showing that, post lockdown, about 56% of participants indicated moderate to high levels of concerns about food, nutrition, and health. While concerns for food supply shortage and undernutrition were low, people were worried about the risks of contracting the virus through contaminated foods or when dining out, as well as about overnutrition. The high prevalence of concern post-lockdown suggested the severity of the mental impacts of the current pandemic and its possible long-lasting effects in reopened areas.

Stresses and concerns may associate with irrational and undesired eating behaviors such as overconsumption of calorie dense foods and extreme eating restrictions, leading to negative health outcomes [32,33]. However, concerns about overnutrition or contracting the virus may also motivate people to pay attention to their diet and eat more healthily to maintain proper weight and protect against chronic diseases and infections. A study among Polish adults reported the existence of two opposite dietary patterns: pro-health and unhealthy, and identified their associated behavioral changes and demographic factors during COVID-19 lockdowns [34]. Indeed, impacts of the COVID-19 pandemic and social-economic restrictions are complex and involve multiple factors that can drive distinctive behavioral changes among diverse populations.

Importantly, our study identified several socio-demographic groups that were more vulnerable to experiencing high levels of food and nutrition related concerns during the COVID-19 pandemic. Older age, being overweight or obese, lower annual household income and educational achievements, and living in urban areas or with vulnerable individuals appeared to increase the level of concern pertaining to food supply, nutrition, and risk of contracting the virus through contaminated food or when eating out. About 34% of our study participants were overweight or obese. Obesity is associated with high levels of stress and concerns about nutrition and health and adverse medical consequences [35,36]. People suffering from obesity are more likely to shift towards unhealthy eating behaviors including emotional and overeating during the current pandemic, which promotes extra weight gain and exacerbates their risks of developing health complications, severe disease cases, and mortality [37,38,39]. On the other hand, while elders are more likely to express concerns about contracting the virus, which is in line with our findings, they are less likely to indicate changes in eating behaviors than younger people during the COVID-19 pandemic [33,36]. Apart from age and weight, characteristics such as socio-economic status and education level can potentially function as important predictors for stress exposures and behavioral changes among different groups of people. Indeed, studies have observed socio-demographic differences among participants’ self-reported alternations in dietary intake, physical activities, sleep patterns, and mental health [8,29,36]. It is therefore critical to identify the nutritional and dietary concerns and needs specific to diverse subgroups and provide appropriate guidance and resources for vulnerable populations.

### 4.2. Grocery Shopping Patterns and Eating Behaviors

Driven by the pandemic and public health measures for COVID-19 prevention and control, the demand for online food shopping, ordering, and delivery has surged worldwide, in particular in Asian countries like China and South Korea where online shopping has expanded and penetrated extensively [14]. Despite the increase in online grocery shopping, purchasing food in person at local supermarkets or small shops remained the predominant channel to obtain food in the post-lockdown period as indicated by our study participants. Several studies observed similar patterns in people’s choices of locations for food purchases during lockdowns [14,15,36]. Certainly, online grocery shopping offers a range of benefits and may reduce one’s exposure to the virus, which is valuable for high-risk populations such as older adults and people with underlying health conditions. Still, some people may find online shopping less favorable due to distrust of previous unpleasant experience; concerns about food contamination during delivery; limited food availability online; technology unfamiliarity and instability; and the need to be outside the house [40,41]. Besides, the reduction in new COVID-19 cases in most areas of China and the reopening of local supermarkets (and therefore increased accessibility to grocery vendors) may encourage people to switch back to shop in store. Further qualitative research in relevant fields is needed to better understand how people make choices for food purchasing (location and frequency) and to explore determinants of health behaviors during and after times of public health crisis.

We report that food and health related concerns have minimum impacts on people’s preference for the locations where they purchase or obtain foods (e.g., online, local supermarkets or small shops, in-house storage, and community food distribution programs) post-lockdown. Modification of habits and behaviors, in particular shopping behaviors, can involve multiple factors such as the built environment, availability and timing of information, and individual motivation, all of which contribute to the determination of behavioral change and formation of various lifestyles [14]. Furthermore, depending on the type of exposure (concerns about food and health in this study) and feasibility of achieving behavioral changes (e.g., knowledge and access to tools and technology), people may respond in similar or distinctive fashions. This is in line with the observation by Di Renzo et al. (2020) who reported a reduction in the frequency of grocery shopping but no significant change in locations during the COVID-19 lockdown among the Italian population [15]. Furthermore, as another study reported and as mentioned earlier, older adults, despite being more concerned about contracting the coronavirus, were more resistant to changes in dietary behaviors during the current pandemic than younger, possibly attributable to stable food shopping and eating patterns and limited alternative options [33].

On the other hand, concerns about food, nutrition, and health may exert positive effects by promoting desired changes in eating and cooking behaviors. Most Chinese people grow up sharing meals with personal chopsticks—an ingrained Chinese dining tradition that shows intimacy around the table. Nonetheless, communal eating may accelerate the transmission of pathogens like coronavirus through saliva [42] and therefore has been advised against by health officials in campaigns such as “dining table revolution” to encourage the use of serving chopsticks and spoons, as well as eating from separate portions rather than from the same plates [43]. We observed that, among our study participants, high levels of food and health related concerns were associated with an increased likelihood of using serving chopsticks, eating from individual plates, and separating plates and utensils for raw and cooked food post-lockdown. Raising concerns and awareness of the risk of contracting COVID-19 and other infectious diseases through communal eating may influence the use of serving chopsticks and other hygiene practices around the dining table to prevent the spread of contagious diseases in Chinese population.

### 4.3. Perceived Change in Food Consumption and Dietary Behaviors Comparing to the Previous Year during the Same Period

Many studies have observed changes in lifestyle and eating behaviors during the pandemic lockdown but very few have explored differences in dietary intake and behaviors comparing the pre-COVID19 era and post-lockdown period as nations reopen and gradually recover from the pandemic. Our data show drastic reductions in the consumption of seafood, raw foods, and imported fresh and raw foods among the study participants compared with the same period last year (2019, before the pandemic). The earliest COVID-19 cases linked to the Wuhan seafood market and recent reports of coronavirus detected on packages of imported seafood might have caused public distrust and discouraged people from consuming seafoods [44,45]. Furthermore, we examined whether some of the dietary changes were mediated by concerns about food, nutrition, and health. The multivariate multinomial logistical regression revealed that people who were moderately or highly concerned about food, nutrition, and health were more likely to eat less seafood, raw foods, and imported fresh and raw foods compared with the same period before the pandemic than people with low levels of concern. In addition, consumption of snack and beverages also reduced among people expressing high levels of food and nutrition related concerns.

Alterations in food choices driven by food and health related concerns may impact individual health and societal well-being during a disease outbreak [11]. Seafoods, for example, are important parts of healthy diets; they are excellent sources of beneficial nutrients like omega-3-fatty acids, protein and minerals with health promoting properties [46,47,48]. However, the COVID-19 pandemic and several cases reported in social media claiming the linkage between imported seafood and local COVID-19 outbreaks in China have disrupted the seafood industry, contributing to global decline in seafood production, trade and consumption and possible long term impacts on populational health and the food system [49,50]. Researchers have also noticed reduction in seafood consumption in other countries such as Kuwait and Spain [13,15]. Policies and evidence-based information are therefore urgently needed to support affected food industries and promote safe and healthy eating where public distrust in certain foods is high. In addition, improving nutrition education among the general public may further benefit informed dietary choices and behaviors to foster and maintain healthy relationships with food.

Noteworthy, similar exposures (e.g., high or moderate levels of food and health related concerns) may induce different behavioral responses and outcomes, partly attributable to variations in individual socio-demographic characteristics and other factors such as personal preference, past experience, and environments. We found that higher levels of concern were associated with both increase and decrease in the consumption of frozen food and purchasing food at farmers’ market or online. People who care and express more concerns about food, nutrition and health may tend to take actions or notice and recall their diet and dietary behavior better than those who do not, hence the increased probability of reporting a change (either decrease or increase). Likewise, Poelman and colleagues conducted a cross-sectional study among adults in Netherland and reported that overweight or obese people—a group of people often concern about their diet—were more likely to indicate following a less healthy diet and also more likely to indicate eating more healthily than normal weight people during lockdown. Similarly, certain food items such as chocolate and wine have been favored and consumed by people in increased amounts to improve mood, but disliked and avoided by people who decided to watch their weight and stay in shape during lockdown [15]. Interestingly, our study showed that being more concerned about food and health seemed to promote the intake of raw food and imported seafood (despite the majority of our study participants indicating decreases consumption of these food items), which might be caused by nutritional concerns or increases in overall food consumption in 2020 comparing to 2019, but more data are needed to confirm the results.

### 4.4. Limitation

This study has several limitations. First and foremost, it was cross-sectional study, relying on self-reports from participants, therefore making it difficult to establish the temporal and causal relationships between food and nutrition related concerns and dietary behavioral changes. The use of an online survey facilitated the distribution and reach of the survey, but it might also have excluded certain populations like those with limited access to the internet, elders, and people who were poorly educated. Moreover, due to the unrandomized nature of the multi-stage sampling method, selection bias may exist. We also noticed that the food shopping locations provided in the survey might not be all inclusive: “farmers’ markets” was not an option in our study survey, but some studies have included it in their questionnaires. Qualitative studies are still needed to understand how public health crises like the COVID-19 pandemic impact people’s perceptions and choices of various food shopping locations.

## 5. Conclusions

Our study showed a high prevalence of food and nutrition related concerns during the post COVID-19 pandemic lockdown period and that these concerns were associated with increased probabilities of reporting changes in dietary behaviors including food preparation and consumption. Current dietary guidelines have limited capacity in addressing dietary concerns during a public health crisis and may lack the specificity to provide nutritional guidance for diverse populations. Several socio-demographic characteristics such as age, weight, and income are associated with higher levels of concern about food, nutrition, and health, suggesting the need for proper programs and policies to support vulnerable populations as well as lifestyle guidance to promote healthy eating. Behaviors and habits formed during lockdowns may continue to exist for an extended period. Both psychological and physical consequences of long episodes of lockdown as well as potential persistence of altered behaviors and their effects on individual and societal health in the post-lockdown and post-pandemic era should be further investigated.

## Figures and Tables

**Table 1:** Food and Nutrition Related Concerns by Socio-demographic Characteristics.

	Concern Scores (Level) ^#^			*p* Values *
	5–8 (L1) *n* = 737	9–11 (L2) *n* = 703	12–20 (L3) *n* = 554	
Gender				
Male (*n* = 742)	39.4	33.6	27.1	0.22
Female (*n*= 1252)	35.5	36.3	28.2	
Age group (year)				
<18	18.0	30.8	51.3	<0.001
18–30	42.3	37.2	20.5	
31–45	33.7	33.1	33.3	
46–60	18.9	29.6	51.5	
>60	10.0	40.0	50.0	
BMI (kg/m^2^) (*n* = 1854)				
Underweight	44.2	34.5	21.3	<0.001
Normal weight	40.4	34.8	24.8	
Overweight	30.5	33.6	35.9	
Obese	25.2	40.4	34.4	
Annual Household income (Chinese yuan)				
<30 k	23.7	25.6	50.7	<0.001
30–100 k	31.7	34.1	34.2	
100–300 k	41.2	39.2	19.6	
300–500 k	48.1	32.0	19.9	
500 k–1 M	46.3	42.1	11.6	
>1 M	44.4	27.8	27.8	
Education				
Less than high school	9.60	24.0	66.4	<0.001
High school graduate	23.5	33.3	43.2	
Bachelors’ degree	39.7	35.2	25.1	
Master’s and higher	45.4	39.7	15.0	
Geographic region				
Urban	39.0	35.6	25.5	<0.001
Rural	27.5	33.8	38.7	
Presence of vulnerable individuals in the same household ^+^				
Yes	32.6	35.4	32.0	<0.001
No	41.3	35.1	23.6	

* *p* values were estimated from Chi-square test; ^#^ Values are displayed in percentages (%); ^+^ Vulnerable individuals include children under the age of 5, elders above the age of 65, and pregnant women.

**Table 2:** Differences of Tendency of Grocery Shopping and Food Storage among Participants with Different Level of Food and Nutrition Related Concerns and Patterns.

	Concern Scores (Level) Differences in Scores from Reference (Mean (SE))				
	5–8 (L1)	9–11(L2)	*p* Value	12–20 (L3)	*p* Value
Online					
Crude ^a^	Ref ^+^	0.10(0.17)	0.55	−0.15(0.18)	0.42
Multivariable-adjusted model ^b^	Ref	0.18(0.17)	0.29	0.17(0.19)	0.38
In-person at supermarket					
Crude ^a^	Ref	−0.08(0.18)	0.66	0.03(0.19)	0.87
Multivariable-adjusted model ^b^	Ref	−0.10(0.18)	0.60	−0.06(0.21)	0.78
Home Food Storage					
Crude ^a^	Ref	0.00(0.19)	0.99	0.33(0.20)	0.09
Multivariable-adjusted model ^b^	Ref	−0.06(0.19)	0.74	0.15(0.22)	0.48
Food distribution programs					
Crude ^a^	Ref	0.07(0.06)	0.22	0.15(0.06)	0.018
Multivariable-adjusted model ^b^	Ref	0.09(0.06)	0.14	0.17(0.07)	0.017

^+^ Ref = Reference group; ^a^ Simple linear regression model (*n* = 1994); ^b^ Linear regression model (*n* = 1854) adjusted for gender, age, education, BMI, annul household income, and geographic regions (urban or rural).

**Table 3:** Associations between Food, Nutrition, and Health Related Concerns Regarding Eating and Cooking Behaviors.

	Concern Scores (Level) Odds Ratio (95% CI)		
	5–8 (L1)	9–11 (L2)	12–20 (L3)
Eating from Individual Plates			
Crude ^a^	1 (Ref)	1.14(0.92, 1.41)	2.12(1.70, 2.65)
Multivariable-adjusted model ^b^	1 (Ref)	1.10(0.88, 1.38)	1.66(1.29, 2.13)
Using Serving Chopsticks			
Crude ^a^	1 (Ref)	1.11(0.90, 1.37)	1.81(1.44, 2.26)
Multivariable-adjusted model ^b^	1 (Ref)	1.13(0.90, 1.41)	1.51(1.17, 1.93)
Cooking Foods Thoroughly			
Crude ^a^	1 (Ref)	1.27(0.72, 2.23)	1.70(0.88, 3.31)
Multivariable-adjusted model ^b^	1 (Ref)	1.42(0.78, 2.59)	1.48(0.73, 3.02)
Separating Plates and Utensils for Raw and Cooked Foods			
Crude ^a^	1 (Ref)	1.14(0.92, 1.41)	1.75(1.37, 2.23)
Multivariable-adjusted model ^b^	1 (Ref)	1.14(0.90, 1.44)	1.78(1.35, 2.36)

^a^ Simple logistic regression (*n* = 1994); ^b^ Logistic regression adjusted (*n* = 1854) for gender, age, education, BMI, annul household income, and geographic regions (urban or rural).

**Table 4:** Frequency of Food Consumption and Dietary Behaviors Comparing to the Same Period in the Previous Year (2019–2020).

		Concern Scores (Level) Relative Risk Ratio (95%CI) ^a^			
	5–8 (L1)	9–11 (L2)	*p* Value	12–20 (L3)	*p* Value
	Food Consumption				
	Seafood				
Increased (*n* = 173)	1 Ref	1.37(0.94, 1.99)	0.10	1.24(0.76, 2.02)	0.39
Decreased (*n* = 989)	1 Ref	1.14(0.90, 1.43)	0.29	1.89(1.44, 2.48)	<0.001
No change (*n* = 692)	Base outcome				
	Raw Food				
Increased (*n* = 63)	1 Ref	0.78 (0.39, 1.53)	0.46	2.24(1.18, 4.27)	0.014
Decreased (*n* = 1127)	1 Ref	1.33(1.06, 1.67)	0.014	1.99(1.52, 2.61)	<0.001
No change (*n* = 664)	Base outcome				
	Frozen food				
Increased (*n* = 348)	1 Ref	1.55(1.15, 2.08)	0.004	1.67(1.17, 2.39)	0.005
Decreased (*n* = 763)	1 Ref	1.34(1.05, 1.72)	0.021	2.50(1.89, 3.31)	<0.001
No change (*n* = 743)	Base outcome				
	Imported Fresh Produce				
Increased (*n* = 61)	1 Ref	1.29(0.69, 2.43)	0.43	2.27(1.13, 4.55)	0.021
Decreased (*n* = 1065)	1 Ref	1.32(1.05, 1.65)	0.016	2.12(1.62, 2.76)	<0.001
No change (*n* = 728)	Base outcome				
	Snack and Beverage				
Increased (*n* = 460)	1 Ref	1.50(1.13, 1.98)	0.005	1.47(1.06, 2.05)	0.022
Decreased (*n* = 705)	1 Ref	1.11(0.86, 1.44)	0.42	1.44(1.08. 1.92)	0.014
No change (*n* = 689)	Base outcome				
	Dietary Behaviors				
	Purchasing Food Online				
Increased (*n* = 778)	1 Ref	1.33(1.05, 1.69)	0.018	1.45(1.00, 2.12)	0.052
Decreased (*n* = 495)	1 Ref	1.19(0.91, 1.57)	0.21	1.83(1.24, 2.69)	0.002
No change (*n* = 581)	Base outcome				
	Purchasing Food at Farmers’ Market				
Increased (*n* = 555)	1 Ref	1.47(1.13, 1.93)	0.005	2.01(1.47, 2.75)	<0.001
Decreased (*n* = 635)	1 Ref	1.53(1.18, 2.00)	0.002	2.18(1.61, 2.94)	<0.001
No change (*n* = 664)	Base outcome				
	Cooking and Eating at Home				
Increased (*n* = 1212)	1 Ref	1.21(0.94, 1.56)	0.14	1.75(1.30, 2.37)	<0.001
Decreased (*n* = 171)	1 Ref	1.17(0.77, 1.80)	0.46	2.20(1.38, 3.53)	<0.001
No change (*n* = 471)	Base outcome				
	Eating Out (e.g., at restaurants)				
Increased (*n* = 224)	1 Ref	1.12(0.77, 1.62)	0.55	1.03(0.66, 1.61)	0.89
Decreased (*n* = 1143)	1 Ref	1.18(0.92, 1.53)	0.20	1.42(1.06, 1.89)	0.018
No change (*n* = 487)	Base outcome				

^a^ Multinomial logistic regression adjusted (*n* = 1854) for gender, age, education, BMI, annul household income, geographic region (urban or rural), and meal regularity.

## Data Availability

Data used in this report may be available upon requests made to the corresponding author.

## Supplementary Materials

The following are available online at https://www.mdpi.com/article/10.3390/foods10112858/s1, A questionnaire on the dietary and nutritional status of residents during the current COVID-19 pandemic.
