# Supplementary material for: Food and Nutrition Related Concerns Post Lockdown during COVID-19 Pandemic and Their Association with Dietary Behaviors
Source: Foods. 2021 Nov 18;10(11):2858. doi: 10.3390/foods10112858 (PMC8624826; doi:10.3390/foods10112858)
Supplement: Supplementary file 1 [file foods-10-02858-s001.zip › supplementary-1.pdf]

新冠疫情常态化居民膳食营养情况问卷调查

您好，我们是北京大学公共卫生学院的学生，为进一步了解此次疫情期间人们膳食多样性和饮食行为，为相关机构提供建议与参考，特进行此次调查。此问卷结果仅供研究使用，绝不会泄露您的个人信息。欢迎您转发此问卷，感谢您的支持与配合！

第一部分:近期食物来源以及过去24小时内摄入食物种类

1.近一个星期食物来源[矩阵多选题] *

	家中原有	前往门店或超市购买	网上购买	政府或社区配送	未食用
a.谷物及谷物类制品（如面包、饼干、米面或其他由小米，大米，高粱，小麦等制成的食物）：	□	□	□	□	□
b.根茎和块茎类食物及其制品（南瓜、胡萝卜、红薯、马铃薯、	□	□	□	□	□

山药等）:					
c.蔬菜:	□	□	□	□	□
d.水果:	□	□	□	□	□
e.肉类、家禽、内脏（比如猪肉、牛肉、羊肉、鸡肉等以及肝脏、肾脏、心脏等内脏肉）:	□	□	□	□	□
f.蛋类:	□	□	□	□	□
g.鱼和海鲜（新鲜鱼类或鱼干，贝类等）:	□	□	□	□	□
h.豆类及制品（如黄豆、豌豆、扁豆等及其制成的食物）:	□	□	□	□	□

i.牛奶和奶制品:	□	□	□	□	□
j.油，脂肪或黄油制品:	□	□	□	□	□
k.糖或蜂蜜:	□	□	□	□	□
l.其他（如速食食品，方便面等，以及代餐零食）:	□	□	□	□	□

2.**过去24小时**内食用食物种类:[矩阵单选题] *

	是	否
是否食用了谷物及谷物类制品（如面包、饼干、米面或其他由小米，大米，高粱，小麦等制成的食物）:	○	○

是否食用了根茎和块茎类食物及其制品（南瓜、胡萝卜、红薯、马铃薯、山药等）:	○	○
是否食用了蔬菜:	○	○
是否食用了水果:	○	○
是否食用了肉类、家禽、内脏（比如猪肉、牛肉、羊肉、鸡肉等以及肝脏、肾脏、心脏等内脏肉）:	○	○
是否食用了蛋类:	○	○
是否食用了鱼和海	○	○

鲜（新鲜鱼类或鱼干，贝类等）：		
是否食用了豆类及制品（如黄豆、豌豆、扁豆等及其制成的食物）：	○	○
是否食用了牛奶和奶制品:	○	○
是否食用了油，脂肪或黄油制品:	○	○
是否食用了糖或蜂蜜:	○	○
其他（如速食食品，方便面等，以及代餐零食）：	○	○

3.如果您是认真作答本题请选是: [单选题] *

○是

○否

第二部分饮食行为

4.近期平均一周运动的次数: [单选题] *

○A.0 (请跳至第6题)

○B.1~3

○C.4~6

○D.7及以上

5.每次运动平均多长时间: [单选题] *

○A.30min以下

○B.30~60min

○C.60min以上

6.近期三餐是否规律: [单选题] *

○一日三餐（时间基本固定）

○一日两餐（时间基本固定）

○一日一餐

○用餐次数、时间不固定

7.近期有无称量体重: [单选题] *

○有称量

○想称，但家里无体重秤

○不想称

8.您目前的身高为：__________ cm
近一个月称量的体重为：_____________kg（公斤）请填整数，若不清楚可填“999” [填空题] *

9.与2019年同期相比，近期您的饮食行为发生的变化：[矩阵单选题] *

	增加	减少	无明显变化
在外就餐频率	○	○	○
通过外卖服务就餐频率	○	○	○
在家自行烹饪就餐频率	○	○	○
通过农贸市场购买食物频率	○	○	○
网上购买食品的频率	○	○	○

食用海鲜频率	○	○	○
生食食品频率	○	○	○
食用冷冻保鲜食品频率	○	○	○
食用进口生鲜频率	○	○	○
食用零食和饮料的频率	○	○	○

10.近一个月以来，您是否注意并坚持以下行为[矩阵单选题] *

	是	否
采用分餐制	○	○
使用公勺公筷	○	○
烹饪时食物煮熟煮透	○	○
生、熟食物的刀具案板分开	○	○

11.请选择您对以下情况的担忧程度[矩阵单选题] *

	毫不担忧	轻微担忧	中度担忧	非常担忧
接触污染食物感染病毒	○	○	○	○
外出聚餐感染病毒	○	○	○	○
食物供应短缺	○	○	○	○
此项请选“非常担忧”	○	○	○	○
营养过剩	○	○	○	○
营养不足	○	○	○	○

12.面对当前疫情形势，您是否有额外摄入以下食品/药品以抵御病毒？ [多选题] *

□维生素C补充剂

□益生菌制剂

□酒或含酒精饮品

□醋

□板蓝根，双黄连等

□食疗补品、保健品

□水果蔬菜

□抗病毒药物

□其他 ________________*

□无额外摄入

13.在当前疫情形式下，您是否增加家中食物的储备量 [单选题] *

○是

○否

14.近一个月以来，您所购买食物是否有以下信息：[矩阵单选题] *

	是	否	未关注
质量检测合格报告	○	○	○
产地来源	○	○	○
运输过程	○	○	○

第三部分 基本信息

15.1.您现在所在的地区: [填空题] *

____

15.2.您所在的地区属于? [单选题] *

○城市

○农村

16.您的年龄为 （岁）？[单选题] *

○18岁以下

○18~30岁

○31~45岁

○46~60岁

○60岁以上

17.您的性别? [单选题] *

○男

○女

18.您的文化程度? [单选题] *

○小学、初中

○高中

○本科

○硕士及以上

19.您的全家年总收入 [单选题] *

○3万以下

○3万-10万

○10万-30万

○30万-50万

○50万-100万

○大于100万

20.近一个月内，您每周出门的次数? [单选题] *

○0次

○1-2次

○3-4次

○5-7次

○大于7次

21.您家中是否有以下人群[矩阵单选题] *

	是	否
5岁以下孩子	○	○
65岁以上老人	○	○
妊娠期妇女	○	○

22.您是否参与本课题组在三月末发放的第一轮问卷？ [单选题] *

○是

○否
