# Supplementary material for: Food and Nutrition Related Concerns Post Lockdown during COVID-19 Pandemic and Their Association with Dietary Behaviors
Source: Foods. 2021 Nov 18;10(11):2858. doi: 10.3390/foods10112858 (PMC8624826; doi:10.3390/foods10112858)
Supplement: Supplementary file 1 [file foods-10-02858-s001.zip › supplementary-2.pdf]

A questionnaire on the dietary and nutritional status of residents during the current COVID-19 pandemic.

Hello, we are students at the School of Public Health of Peking University. We are conducting a survey to further understand the dietary diversity and behaviors of people during the current COVID-19 pandemic, and to provide references and recommendations for relevant institutions. The results of this questionnaire are for research use only and will never reveal your personal information. You are welcome to forward and share this questionnaire and thank you for your support and cooperation!

Section 1: recent food sources and types of food consumed within the past 24 hours

1. food source in the past week [matrix multiple-choice] *

	Home food storage	In-person shopping at supermarkets	Online purchase	Food distribution programs	Did not consume
a. Cereals and cereal products (such as bread, biscuits, rice noodles or other foods made from millet, rice, sorghum, wheat, etc.):	◻	◻	◻	◻	◻
b. Root and tuber foods and their	◻	◻	◻	◻	◻

products (pumpkin, carrot, sweet potato, potato, yam, etc.):					
c. Vegetables:	□	□	□	□	□
d. Fruits:	□	□	□	□	□
e. Meat, poultry, organ meat (such as pork, beef, lamb, chicken, etc., as well as liver, kidney, heart and other organ meat):	□	□	□	□	□
f. Eggs:	□	□	□	□	□
g. Fish and seafood (fresh fish or dried fish, shellfish, etc.):	□	□	□	□	□

h. Beans and products (Such as soybeans, peas, lentils and foods made from them etc.):	□	□	□	□	□
i. Milk and dairy products:	□	□	□	□	□
j. Oil, fat or butter and their products:	□	□	□	□	□
k. Sugar or honey:	□	□	□	□	□
l. Other (such as instant food, instant noodles, snack and meal replacement):	□	□	□	□	□

2. Types of food consumed in the past 24 hours: [multiple choice matrix] *

	Yes	No

a. did you consume cereals and cereal products (such as bread, biscuits, rice noodles or other foods made from millet, rice, sorghum, wheat, etc.):	○	○
b. did you consume root and tuber foods and their products (pumpkin, carrot, sweet potato, potato, yam, etc.):	○	○
c. did you consume any vegetables:	○	○

d. did you consume fruits:	○	○
e. Meat, poultry, organ meat (such as pork, beef, lamb, chicken, etc., as well as liver, kidney, heart and other organ meat):	○	○
f. did you consume eggs:	○	○
g. did you consume fish and seafood (fresh fish or dried fish, shellfish, etc.):	○	○
h. did you consume beans and products	○	○

(Such as soybeans, peas, lentils and foods made from them etc.):		
i. did you consume milk and dairy products:	○	○
j. did you consume oil, fat or butter and their products:	○	○
k. did you consume sugar or honey:	○	○
l. did you consume other foods (such as instant food, instant noodles, snack and meal	○	○

replacement)<br>:		

3. If you are serious about answering this question, please choose yes: [single-choice question] *

○ yes

○ no

Section 2: eating behaviors

4. Recently, on average, how many times you have performed any physical activities in a week? [single-choice question] *

○A.0 (please skip to question 6)

○B.1~3

○C.4~6

○D.7 and above

5. What is the average duration (in minutes) each time you exercise? [single choice] *

○A. 30min or less

○B.30~60min

○C.60min or more

6. How regular your meals have been recently? [multiple choice] *

○Three meals a day (mostly fixed time)

○Two meals a day (mostly fixed time)

○ One meal a day (mostly fixed time)

○ No fixed meal frequency and time

7. Have you weighed yourself recently? [Single-choice question] *

○ Yes

○ Want to but no scale available at hom

○ No, do not want to weigh

8. Your current height is: _________ cm

Your weight in the past month is: ____________kg (kilograms) [fill in the blank] *

Please use integers.

If not known, please fill in "999"

9. compared with the same period in 2019, please report any recent changes in your eating behaviors: [matrix multiple choice] *

	increase	decrease	no significant change
Frequency of eating out	○	○	○
Frequency of ordering takeout or delivery	○	○	○

Frequency of cooking and eating at home	○	○	○
Frequency of buying food at the farmer’s market	○	○	○
Frequency of buying food online	○	○	○
Frequency of eating seafood	○	○	○
Frequency of eating raw foods	○	○	○
Frequency of eating frozen foods	○	○	○
Frequency of eating	○	○	○

imported foods			
Frequency of snacking and drinking f	○	○	○

10. In the recent month, have you paid attention and adhered to the following behaviors: [matrix multiple choice] *

	Yes	No
Eating from individual plates	○	○
Using public chopsticks	○	○The
Cooking foods thoroughly	○	○
Separating plates and utensils for raw and cooked foods	○	○

11. Please select the level of your worries about the following situations [matrix single-choice question] *

	not at all concerned	slight concerned	Moderately concerned	Extremely concerned

contracting coronavirus through contaminated foods	○	○	○	○
contracting coronavirus when 120 dining out	○	○	○	○
Shortage of food supply	○	○	○	○
Please select "extremely concerned" for this item	○	○	○	○
Overnutrition	○	○	○	○
Undernutrition	○	○	○	○

12. During the current pandemic, have you consumed more of the following foods/medicines to protect against the virus? [Multi-choice questions] *

□Vitamin C supplements

□Probiotics

□Alcohol or alcoholic beverages

□Vinegar

□Banlangen, Shuanghuanglian etc.

□Dietary supplements or health care products

□Fruits and vegetables

□Antiviral drugs

□Other_________________*

□No additional Intake

13. During the current pandemic, have you increased the amount of food storage at home? [single choice] *

○Yes

○No

14. In the past month, did the foods that you purchased display any of the following information: [Matrix single-choice question] *

	Yes	No	No issue
Quality inspection report	○	○	○
Origin	○	○	○
Transportation	○ ○		○

Section 3: basic demographic information

15.1 where are you currently located?(equivalent to county level residence): [fill in the blank] *

____

15.2 this location is: Multiple-choice questions] *

○urban

○rural

16. What is your age (in years)? [Single-choice questions] *

○under 18 years old

○18-30 years old

○31~45 years old

○46~60 years old

○over 60 years old

17. Your gender? [single-choice question] *

○male

○female

18. What is your education level? [single-choice question] *

○ elementary school or junior high school

○ high school

○ bachelor’s degree

○ master's degree and above

19. What is your annual household income (in Chinese Yuan)? [single choice title] *

○ 30 thousand or less

○ 30 to 100 thousand

○ 100 to 300 thousand

○ 300 to 500 thousand

○ 500 thousand to 1 million

○ more than 1 million

20. **in the past month**, **how many times did you go outside?** [Multiple choice] *

○ 0 times

○ 1-2 times

○ 3-4 times

○ 5-7 times

○more than 7 times

21. In your household, are there any: [matrix multiple choice] *

	Yes	No
children under 5 years	○	○
people over 65 years old	○	○
pregnant women	○	○

22. Did you participate in the first round of survey distributed by this research group at the end of March 2020? [Single-choice question] *

○Yes

○No
